# Causal relationship between local field potential and intrinsic optical signal in epileptiform activity *in vitro*

**DOI:** 10.1038/s41598-019-41554-x

**Published:** 2019-03-26

**Authors:** Zsigmond Benkő, Kinga Moldován, Katalin Szádeczky-Kardoss, László Zalányi, Sándor Borbély, Ildikó Világi, Zoltán Somogyvári

**Affiliations:** 1 Theoretical Neuroscience and Complex Systems Research Group, Department of Computational Sciences, Wigner Research Center for Physics of the Hungarian Academy of Sciences, Budapest, H-1121 Hungary; 2 János Szentágothai Doctoral School of Neurosciences, Semmelweis University, Budapest, H-1085 Hungary; 3 Department of Physiology and Neurobiology, Eötvös Loránd University, Budapest, H-1117 Hungary; 4 Neuromicrosystems ltd., Budapest, H-1113 Hungary

## Abstract

The directed causal relationship were examined between the local field potential (LFP) and the intrinsic optical signal (IOS) during induced epileptiform activity in in vitro cortical slices by the convergent cross-mapping causality analysis method. Two components of the IOS signal have been distinguished: a faster, activity dependent component (IOSh) which changes its sign between transmitted and reflected measurement, thus it is related to the reflectance or the scattering of the tissue and a slower component (IOSl), which is negative in both cases, thus it is resulted by the increase of the absorption of the tissue. We have found a strong, unidirectional, delayed causal effect from LFP to IOSh with 0.5-1s delay, without signs of feedback from the IOSh to the LFP, while the correlation was small and the peaks of the cross correlation function did not reflect the actual causal dependency. Based on these observations, a model has been set up to describe the dependency of the IOSh on the LFP power and IOSh was reconstructed, based on the LFP signal. This study demonstrates that causality analysis can lead to better understanding the physiological interactions, even in case of two data series with drastically different time scales.

## Introduction

Different methods are used to monitor activity-dependent changes in nervous tissue, when both neuronal and glial cell function may be altered. Beside detection of fast electrical changes with different electrophysiological approaches, optical techniques can also be used to follow neuronal activity with a non-invasive manner^1,2^. The intrinsic optical signal (IOS) quantifies the changes of optical properties of the tissue by measuring the transmitted or the reflected light in brain slices. Contrary to the *in vivo* IOS method, the *in vitro* IOS is independent of the blood flow changes, and it may develop as a result of different cellular processes, such as ionic movement across the membranes, synaptic activity and metabolic changes, which may cause cell swelling or shrinkage^3^. These volumetric changes of both neural and glial cells lead to changes in the light scattering properties of the tissue, thus it is widely accepted, that they form the basis of the IOS generation: swelling of the cells decreases the scattering, thus increases the transmittance of the tissue^1–5^. Although most of these processes are slow, there are evidences of faster IOS components as well, which may change on time scales similar to the electrophysiological signals. While the correlation between electrical- and intrinsic optical signals has been known for a long while^4,6^, the precise causal relationship of electrical and intrinsic optical signals has not yet been analyzed in detail. In spite of several studies, it is not yet clarified, how different IOS components parallel develop, and it is also questionable, which types of underlying processes are consecutively activated^7^. Epileptiform activity can be easily provoked in acute brain slices in various ways. Different chemicals, i.e., 4-aminopyridine (4AP) applied into the incubation solution results in spontaneous epileptic discharges in hippocampal or cortical brain slices^8^. In *Mg*^2+^-free solution, spontaneous epileptiform activity develops with slightly different characteristics^9^. Strong electrical stimulation of the white matter results in after-discharges, which can be regarded as an electrically evoked epileptiform activity^10^. The aim of our present investigation was to analyze the processes of IOS generation following seizure induction in acute brain slices, when both fast and slow IOS components are detectable^11^.

The correlated appearance of epileptiform electrophysiological and IOS signals raised the question of the direction and type of the causal connection between them. At least two alternative hypothesis can be set up for the causal relation between the activity observed in the local field potential (LFP) and the activity dependent IOS components: On one hand, it is possible that the epileptiform activity causes the osmotic changes, and the resulting swelling of the cells is measured by the IOS, while the electrophysiological activity develops and runs independently from those osmotic changes. In this case a unidirectional causal drive would exist from the LFP to the IOS. On the other hand, it is also possible that the changes within the ionic concentration, reflected by the IOS signal, have a feedback effect on the epileptic activity, presumably determining the termination and the recurrence of the epileptic bursts. In this case, circular or bidirectional causal connection would exists between the LFP and the IOS signals.

As the new causality analysis method called convergent cross-mapping (CCM), introduced by Sugihara *et al*.^12^ and extended to delayed causal effects by Ye *et al*.^13^, is well suited to determine both unidirectional and circular causal couplings, we applied this method in this study, to determine the causal relationship between the electrophyisiological and optical signals.

## Results

**Figure 1:**
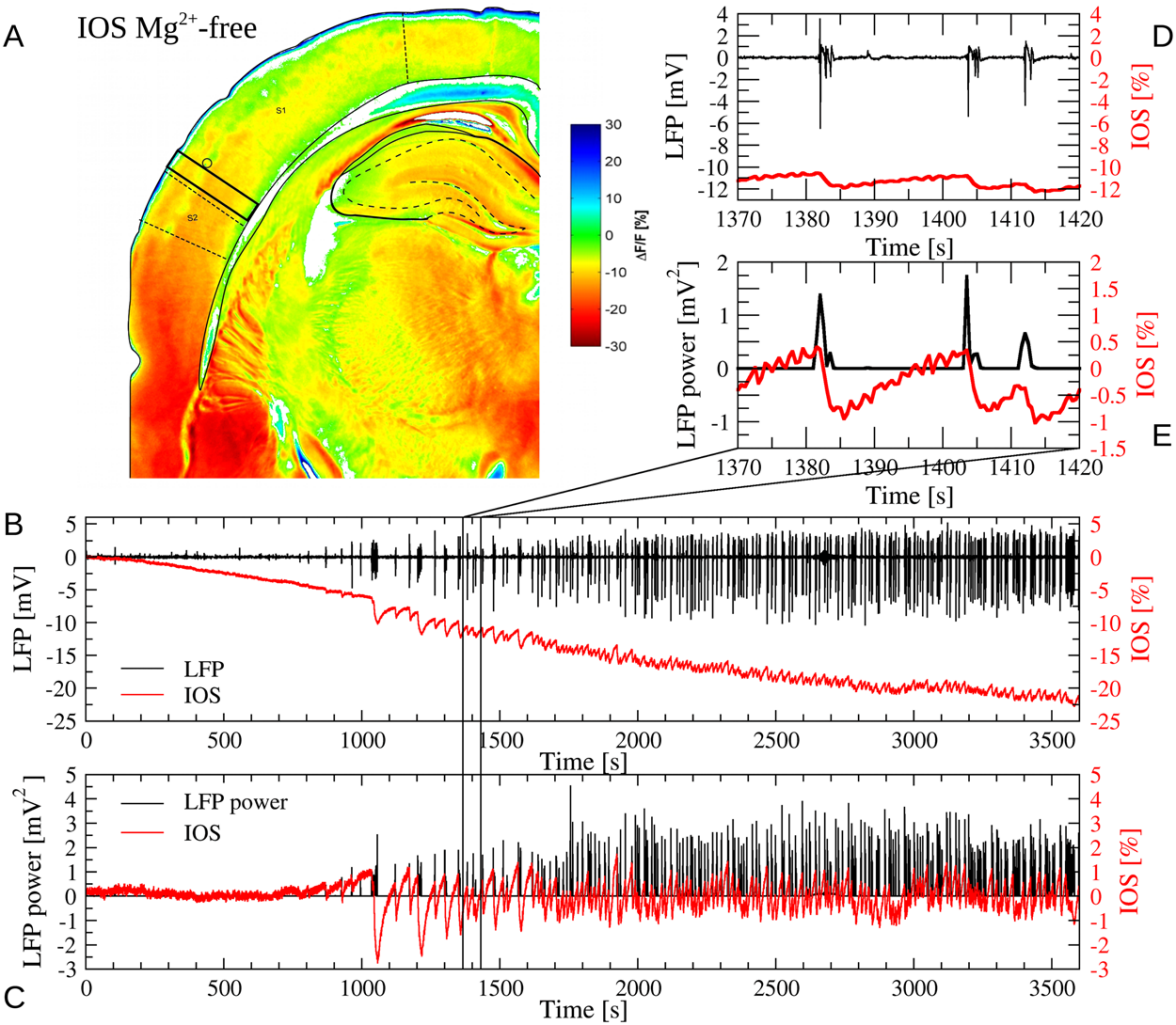
The spatial distribution and temporal evolution of the IOS. (**A**) Pseudo color plot of the relative reflectance changes (IOS) of the coronal brain slice. The temporal evolution of the IOS was tracked by calculating the mean IOS within the ROI in the somatorensory cortex (rectangle with solid black line) close to the electrode position (open circle) in which the LFP was recorded. (**B**,**D**) Parallel recorded LFP (black) and the IOS (red) during epileptiform activity induced by *Mg*^2+^-free artificial cerebrospinal fluid. (**C**,**E**) Downsampled LFP power (black) and detrended IOSh (red) are the variables for which the causality was analyzed.

Three methods have been applied to evoke the epileptiform activity in rat neocortical slice preparations: electric stimulation (STIM, 5 slices), application of *Mg*^2+^-free artificial cerebrospinal fluid (4 slices) and 4-aminopiridine (4AP) treatment (4 slices). All the three methods evoked epiletiform activity by means of recurrent population burst discharges in the slices. There were specific differences between the burst discharges which were characteristic for the method of induction and there were large variation among slices as well. The induction-specific characteristic properties include the amplitude, the intraburst and interburst frequencies as well as the temporal development of the activity during the 1 hour long measurements^14^. The IOS was measured parallel, by quantifying the relative changes in the reflected or transmitted light flux over the somatosensory cortex. In 12 slices, the IOS signal was measured in interface setup, which allowed the measurement of the reflected light, but in one slice, the *Mg*^2+^-free induced activity was measured in submerged setup, which made possible the measurement of the transmitted light. An example of the *Mg*^2+^-free evoked epileptiform activity, the parallel recorded LFP and reflected IOS are shown in Fig. 1B.

### The slow and the fast components of the IOS signal

**Figure 2:**
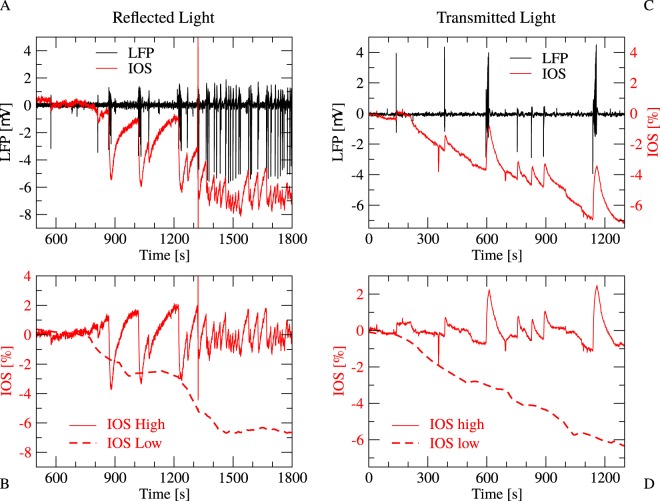
Comparison of the IOS activity in reflected and transmitted light during epileptic activity evoked by *Mg*^2+^-free solution (**A**) LFP (black) and IOS (red) signal in reflected light. (**B**) High (IOSh, solid line) and low (IOSl, dashed line) frequency components of the IOS signal. IOSl was calculated by moving window averaging the IOS time series, while IOSh is a result of the subtraction of IOSl from IOS. (**C**) LFP (black) and IOS (red) signal in transmitted light. (**D**) IOSh and IOSl in transmitted light. Note, that IOSl were negative in both the reflected and the transmitted measurements, while the negative IOSh changed its sign in the transmitted setup.

The general temporal development of the reflected IOS signal during the epileptic activity induction is consists of two components: The first component is a slow and slowly saturating negative shift in the baseline, while the second, faster component takes shape in negative waves descending after each epileptic discharge and slowly converging back to the baseline during the discharge-free periods (Fig. 1). Comparing the IOS time courses in transmitted and reflected light, we found that the slow component is negative in both cases, while the faster component changed its sign: negative in reflected, but positive in the transmitted light (Fig. 2A,C). Considering, that scattering decreases the flux of the transmitted light but increases the amount of the reflected light, while absorption decreases both the transmitted and the reflected components, we concluded, that the two components differ not only in their time scale, but also in the underlying mechanisms. The slow component is a result of the absorption of the tissue, while the faster component corresponds to the changes in the scattering or reflectance of the neural tissue. Thus, we divided the two signals by subtracting a 100 s long moving window average from the raw IOS, resulting in a slower, low frequency component IOSl and a faster, high frequency component IOSh signal (Fig. 2B,D).

### Time delayed causality between LFP power and the fast component of IOS

The sampling frequency of the LFP signal was 1 kHz, while the sampling rate of the parallel IOS recordings were only 2 Hz. As the dynamics of IOS is much slower than the LFP, this low sampling rate was enough to track the temporal changes of the IOS. However, the causality analysis requires fully synchronous time series. To reach this, the LFP signal was undersampled, by calculating the sum of the squared LFP amplitudes (the LFP power) within each 0.5 s long sampling interval.

**Figure 3:**
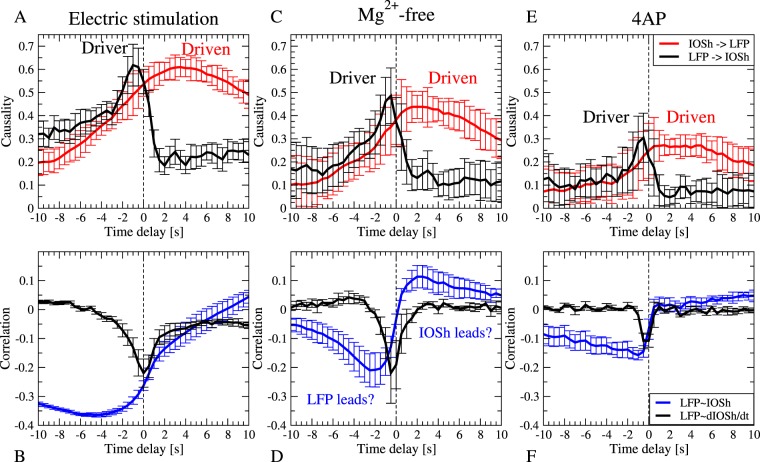
Causality as a function of time delay during epileptiform activity evoked by three different forms of evoked activity. (**A**) Epileptiform activity is evoked by electrical stimulation. The mean cross-map function and the SE over the slices (n = 5) are shown. The LFP clearly drives the IOS with short time delay: the causality peak is observed at 1 s delay (black line). The causality peak in the IOSh → LFP direction with positive (anti-causal) time delay shows delayed correlation: the IOSh follows the LFP with 3 s delay (red line). (**B**) Cross-correlation function between LFP and IOSh (blue line) and between the LFP and the time derivative of the IOSh (black line). The peaks of the LFP-IOSh cross-correlation function does not corresponds to the causality peaks, while the derivative of the IOSh correlates with the LFP at 0 time lag, which is closer to time lag of the causality. (**C**,**D**) Same as in (**A**,**B**), but in epileptic activity was evoked by *Mg*^2+^-free artificial cerebrospinal fluid, mean and SE (n = 3). Similarly, the causality analysis implies unidirectional effect: the LFP causes the IOSh, but with only 0.5 s time delay, which corresponds to the negative peak of the cross-correlation function between the derivative of the IOSh and the LFP power (**D** black line). (**E**,**F**) Same as (**A**,**B**), but epilepsy was evoked by 4-aminopyridyn (4AP) mean and SE (n = 4). Both the causality and the correlation peaks are smaller but their temporal relations are the same. The causality peak coincides with the correlation peak between the LFP and the derivative of the IOSh.

The CCM method of Sugihara^12^, extended for delayed effects by Ye *et al*.^13^, was implemented in Scilab and applied to reveal the causality between the 1 hour long LFP and the IOSh recordings. Applying the time-delayed CCM analysis to the downsampled LFP power and to the IOSh recordings from each slice in the reflected light setup, we constructed the cross-map functions, which express the strength of the causal connections in both directions, as a function of the time delay between the two time series (Fig. 3). The resulting cross-map functions were averaged for slices sharing the same induction process: electric stimulation, *Mg*^2+^-free solution and 4AP treatment.

The causality analysis revealed similar structure in all the three cases. The cross-map functions have two significant peaks: a sharper peak for the LFP → IOSh causal direction, where the LFP precedes the IOSh with 1 s in case of electric stimulation and with 0.5 s in *Mg*^2+^-free and 4AP cases (Fig. 3A,C,E black lines, peaks on the negative half of the x axis) and a wider peak in the IOS → LFP direction, where IOSh follows LFP with 2.5 s time delay in the electric stimulation case and 1.5 s delay in the *Mg*^2+^-free and 4AP cases (Fig. 3A,C,E red lines, peaks on the positive half of the x axis).

In all cases, the peaks of the IOSh → LFP causality function are located at an anti-causal time delay, which means, that the presumed cause, the IOSh, in these cases, follows the presumed effect (the LFP) in time. Assuming that there is no difference in the observational delay, this is clearly impossible if the causal premise is correct. But, how can these anti-causal peaks be interpreted? Sugihara’s CCM search for the fingerprints of the cause in the caused time series, thus a delayed CCM peak, means that the past cause can be reconstructed best from a later section of the caused time series. In our case, the real cause, the past LFP, can be reconstructed from the IOSh 0.5–1 s later. In the reverse (IOSh → LFP) direction, the algorithm checks, if the IOSh can be determined from the LFP. Thus the anti-causal peaks in the IOSh → LFP direction mean that the future of the IOSh can be determined based on the earlier LFP. We expect the appearance of such an anti-causal peak if the caused dynamics are more deterministic and non-chaotic, thus their future can be determined from the cause for a long time.

Thus, the conclusion of the causality analysis is that the LFP drives the IOSh activity, which follows quite predictably the LFP dynamics. There were no significant peaks on the negative half axis of the cross-map functions in the IOSh → LFP direction, which means that we did not find evidence for a feedback effect from the IOSh to the LFP in the examined range of time delays.

As the cross-map functions showed similar structures in all the three cases, we can assume that although the mechanisms generating the epileptic activity are different in the three cases, the causal link, thus the underlying mechanism, connecting the LFP to the IOSh may be similar.

Theoretically, the cross-map between two fully deterministic systems should reach the amplitude 1 for data series long enough. Thus, maxima smaller than 1 could be the effect of noise (which is non-deterministic) and that the recordings are not long enough. We found, that the amplitude of the causality peak was highest for the electrical stimulation case (mean 0.62 ± 0.09 SE), smaller for the *Mg*^2+^-free (mean 0.44 ± 0.16 SE) and smallest for the 4AP evoked activity (mean 0.3 ± 0.11 SE). The difference within the amplitudes could be the result of the different activity level (less discharges) within the cases as well as the different signal to noise ratio.

## Causality versus correlation

We have compared the cross-map function to the traditional cross-correlation function. The cross-correlation function between LFP power and IOSh also showed similar structure in all cases, albeit with some differences (Fig. 3B,D,F blue lines). All the three correlation functions had a significant negative peak on the negative half axis (corresponding to the LFP lead) although at different delays: at −4.5 s in case of electric stimulation (mean −0.36 ± 0.007 SE), at −2.5 s in the *Mg*^2+^-free case (mean −0.21 ± 0.06 SE) and at −1 s in the 4AP case (mean −0.16 ± 0.016 SE). The cross-correlation function showed a second significant but positive peak on the positive half axis only at the *Mg*^2+^-free case (mean −0.11 ± 0.004 SE), but increased monotonically in the two other cases. Considering, that for very large time delays, the two data series become independent, thus the cross-correlation function should decay to zero, this monotonic increase implies the existence of a second even wider positive peak shifted far into the positive direction in these cases as well.

Comparison of the cross-correlation functions (Fig. 3B,D,F blue lines) to the LFP → IOSh causality function (Fig. 3A,C,E black lines) shows, that none of peaks of the cross-correlation functions corresponds to the real delay of the causal effect represented by the peaks of the cross-map functions. We concluded that the real causal effect is not clearly reflected in the peaks of the cross-correlation functions in these cases.

As the short delay (*δ* < 0.5 s^−1^ s) causal effect from the LFP to the IOSh is not accompanied by high correlation with similar short delay, the effect from the LFP to the IOSh should take a form, which can not be well approximated with the linear dependence. One possibility is that, instead of the IOSh, the derivative of the IOSh depends on the LFP power. This assumption is supported by the shape of the cross-correlogram: highest steepness is close to zero time delay.

Thus, we also calculated the cross-correlation function between the LFP power and the time derivative of the IOSh signal. We found, that the peaks of the causality (Fig. 3A,C,E black lines) well matches to the negative peaks of the LFP-dIOSh/dt cross-correlation function (Fig. 3B,D,F black lines) in all the three cases. This implies the possibility, that the temporal derivative of the IOSh depends on the LFP power during the epileptiform discharges in the tissue.

## Autonomous dynamics of IOSh without discharges

**Figure 4:**
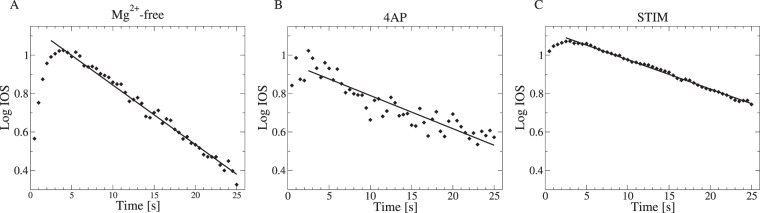
Three examples of the exponential decay of IOSh mean absolute value between discharges. The mean normalized IOSh amplitudes after initiation of epileptiform bursts at 0 s on Lin-Log plots (diamonds) and the fitted exponentials (solid lines) are shown. In all the three cases the decay closely followed the exponential rule between 2.5 and 25 s. The characteristic decay time of the fitted exponentials in these examples were (**A**) 32.3 s in the *Mg*^2+^-free case; (**B**) 58 s in the 4AP case and (**C**) 65.62 s in the electrically stimulated case.

According to the above results, a causal link clearly exists between LFP and IOSh. However, between the burst discharges, the IOSh decays towards the baseline, which, in lack of observable electric activity, seems to be a result of autonomous dynamics (Figs 1E and 2). In order to determine the form of the decay functions, we collected the IOSh time series during those interburst periods which were longer than 25 s from all slices. The amplitude of the IOSh was normalized to 1 for each period and averaged over all bursts within a slice. The logarithm of the absolute value of the IOSh showed clear linear temporal dependence, during large part of these interburst periods in all cases, which is the hallmark of exponential decay dynamics. Thus, a linear function was fitted to the logarithm of the mean IOSh values between 2.5 and 25 s for each slices. Finally the mean and the SD of the decay time constant were calculated for the three forms of the epileptic activity. The mean decay time constant was the shortest in the *Mg*^2+^-free evoked activity with *τ*_1_ = 30.3 ± 3.2 s (11, 9 and 11 intervals from 3 slices); the decay was longer in the 4AP treated slices: *τ*_1_ = 54.9 ± 18.7 s (15, 7, 7, 11 intervals from 4 slices) and the longest for the electrically stimulated slices: *τ*_1_ = 73 ± 19.2 s (25, 25, 6, 15, 6 intervals from 5 slices). Three examples of the exponential fits are shown in Fig. 4, one for each of the three forms of evoked epileptic activity.

## Reconstruction of IOSh based on LFP

By combining these observations, a simple formula is inferred, approximately describing the dependency of the IOSh on the LFP power in the form of a differential equation:

1 $$\frac{dIOSh}{dt}=W(t)\,\ast \,LF{P}^{2}(t)-\frac{IOSh}{{\tau }_{1}}$$

where W(t) scales the causal effect from LFP to IOSh. Here we assumed, that the decay constant *τ*_1_ is constant through whole recordings. This simple model has been used to estimating the IOSh signal based on the known LFP power in the *Mg*^2+^-free and 4AP elicited cases, where the epileptiform activity has developed autonomously, without external stimulation. The reconstructed IOSh were filtered similarly by subtracting the moving window average as the real observed signal and were compared to it. The optimal model parameters were determined by numerical optimization, minimizing the Root Mean Square Error (RMSE) of the IOSh reconstruction.

First, W(t) was set to a constant value according to the negative peak of the correlation between LFP power and the time derivative of the IOSh, but we found, that the residual error of the IOSh reconstruction showed clear temporal tendency in majority of the recordings. Thus we introduced a time dependent weight factor W(t) in form of an exponential decay:

2 $$W(t)={W}_{0}{e}^{-\frac{t}{{\tau }_{2}}}$$

The optimal *W*_0_ and the *τ*_2_ time constant was determined by a grid search. The optimization converged to a minimum of the RMSE in 6 cases out of the 7 slices (2 out of 3 *Mg*^2+^-free and all the 4 4AP slices). Thus we concluded, that the exponentially decaying effective connection strength was a reasonable description of the long term temporal development in the majority of the cases. The optimal time constant were found to be *τ*_2_ = 2839 ± 582 s (mean and SD) for the *Mg*^2+^-free cases and *τ*_2_ = 2061 ± 1121 s (mean and SD) for the 4AP cases.

**Figure 5:**
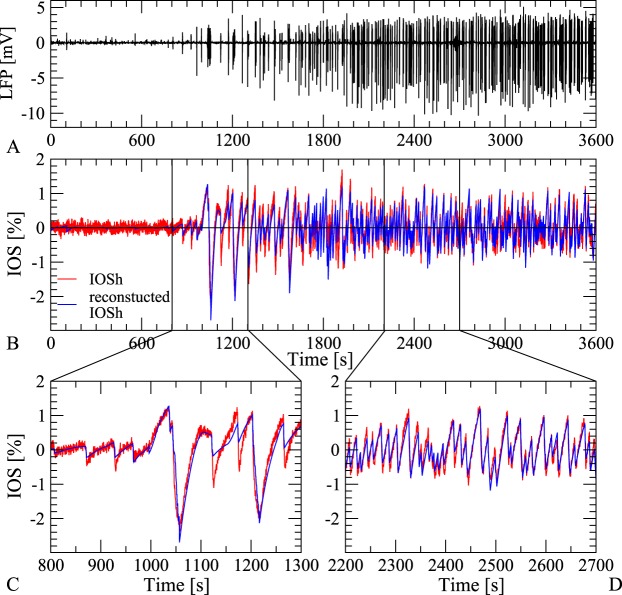
Reconstruction of IOSh based on LFP in *Mg*^2+^-free case. (**A**) The recorded LFP signal showing the epileptiform activity induced by *Mg*^2+^-free solution (black). (**B**) Comparison of the high pass filtered IOS (red) and the reconstructed IOSh (blue) based on the LFP signal. (**C**,**D**) Zoom of the original and reconstructed IOSh signal in the early and late phase of the epileptic activity. Our simple model reconstructs the IOSh signal based on the LFP with high precision, through the 1 hour long experiment.

**Figure 6:**
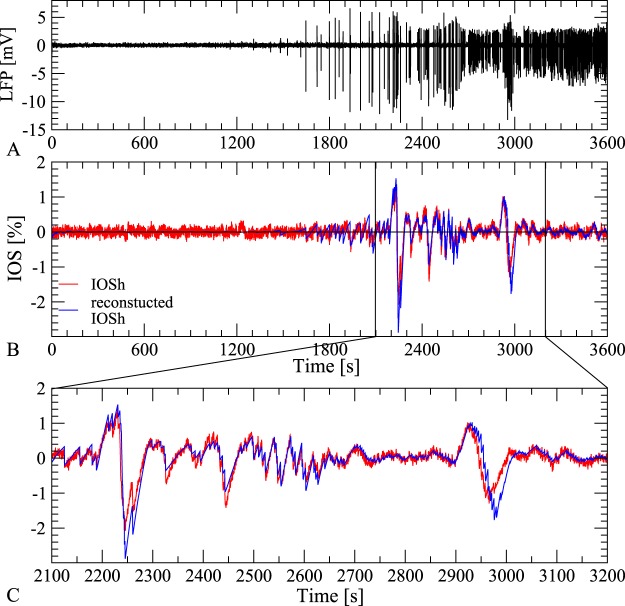
Reconstruction of IOSh based on LFP in 4AP case. (**A**) The recorded LFP signal showing the epileptiform activity induced by 4AP treatment (black). (**B**) Comparison of the high pass filtered IOS (red) and the reconstruction (blue) based on the LFP signal. (**C**) Zoom of the original and reconstructed IOSh signal shows clear dynamical changes, but our simple model reconstructs the IOSh signal based on the LFP with high precision, through the 1 hour long experiment.

The comparisons between the reconstructed and the observed IOSh show that reconstruction follows closely the actual observations during the 1 h long experiment, through several dynamical changes in both the *Mg*^2+^-free and the 4AP cases. An example for *Mg*^2+^-free elicited activity is shown in Fig. 5 and another for 4AP in Fig. 6. Note, the large difference between the two dynamics and waveshape.

### Simulations

Based on the derived formula for the LFP → IOSh dependence, simulations have been run in order to cross-validate the causality analysis results.

The simulations consist of a driving variable (*X*), a logistic chaotic oscillator, from which a spike-like discharge activity is derived, by raising it to the 4^*th*^ power, and a second driven variable exhibiting linear dynamics and exponential decay corresponding to the inferred model of the IOSh:

3 $$X(t+\mathrm{1)}=3.8X(t)\,\mathrm{(1}-X(t))$$

4 $$IOSh(t+\mathrm{1)}=\mathrm{(1}-dt/{\tau }_{1})IOSh(t)-{X}^{4}(t-\delta )$$

**Figure 7:**
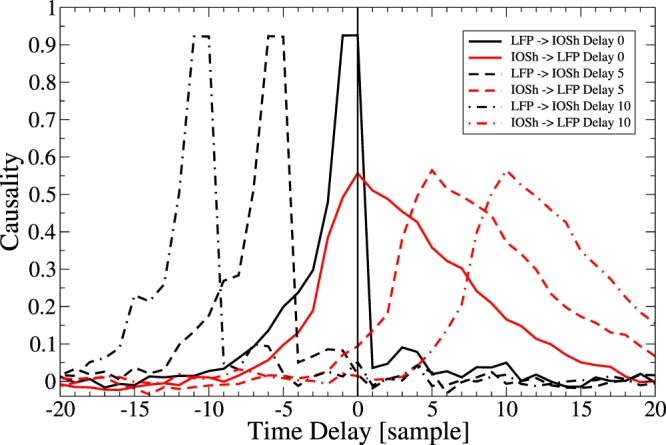
Test of causality analysis on simulated test data. Simulated IOSh have been driven by the simulated LFP with three different time delays in the three cases: *δ* = 0, 5 and 10 time samples. The positions of the peaks of the black lines (solid, dashed and dash-dot) on the negative half of the x axis clearly mark both the direction and the delays of the driving force. The peaks of the red lines at positive delay values show, that the IOSh follows predictably the LFP.

The time delay *δ* of the driver effect have been varied and the causal relationship have been measured by the cross-map function. These simulations showed, that the exact value of the time delay between the two variables was inferred precisely by the peaks of the cross-map function, and even the shape of the cross-map function and the anti causal peak in the IOSh → LFP direction resembles very much to the observed one (Fig. 7). Based on these simulations, we can conclude that the time delay of the causal effect can be determined precisely by this method, in this case.

## Discussion

Two components of the IOS signal have been distinguished during induced epileptic activity in *in vitro* cortical slices. They were different not only in their time scale, but presumably the underlying mechanisms as well. The faster, activity-dependent component (IOSh) was positive in transmitted light and negative in reflected light measurements. It can be interpreted as the decrease of the scattering of the tissue, caused by swelling of the cells due to the activation and underlying movement of ions across the membranes followed by water^15^. In this processes not only the neurons but also the glial cell might play significant role^16,17^. The later types of cells are more prone to swell, but the glial reaction is preceded by neuronal activation^18^. During this activation, an abundance of excitatory transmitters are released and intensive ionic movements take place increasing significantly the extracellular *K*^+^ concentration, which is then buffered in glial cell and causes its swelling^19^.

The slower component (IOSl), however was negative in both transmitted and reflected experiments, thus it can be attributed to the increase of the absorption of the tissue. While different components of the *in vitro*, blood-free IOS signal were distinguished previously^1,5,20^ all of them were attributed to the change of the scattering of the tissue, thus none of them corresponds to the observed increase of the absorption. Cell death could be one of the possible reasons of this phenomena, however, as the electrical responsiveness of the slices haven’t changed significantly during the recording, we can suppose, that cellular destruction is not necessarily substantial. In order to verify the possible role of the cell death in the IOSl component, its irreversibility should be checked.

A candidate mechanism, showing that absorption plays role in IOS generation, was presented by Mané and Müller^21^: Multispectral analysis of the IOS showed a significant dip in the IOS spectrum at 440 nm wavelength during spreading depression. This absorption line, referred to as Soret band, is a mark of the absorption of porphyrins. The changes of the light absorption by cytochromes appear at this wavelength, thus, reduction of cytorchromes as well as its unpacking from the mitochondria could increase the absorption of the tissue.

The directed causal relationships were examined between the LFP and the IOSh by Sugihara’s causality analysis method, the convergent cross mapping. We have found a strong, unidirectional, delayed causal effect from LFP to IOSh with 0.5–1 s delay, without signs of feedback from the IOSh to the LFP. However, it is also showed, that a “shadow peak” in the IOSh → LFP direction appeared at anticausal time delays.

In general, we found, that the delay of the causality peaks significantly affects the interpretation of the results. Assuming no observational delay, two different cases should be distinguished: the peak of the cross-map functions, located on the negative half of the time axis, should be considered as a sign of the real causal effect, since the cause preceded the consequence. However, the peaks on the positive half-axis should be interpreted, as a sign of the delayed prediction, ie. the caused time series follows the effect of the cause faithfully with some delay, thus not only the cause can be reconstructed from the caused time series (this corresponds to the peaks on the negative axis), but the caused time series can be predicted from the cause as well. While the peaks on the negative half of the delay axis are signs of the causality in terms of the Sugihara *et al*.^12^, the peaks on the positive half-axis correspond more to the predictive causality according to the Wiener-Granger principle^22–24^.

In our case, due to the relatively smooth and continuous nature of the IOSh signal, the IOSh → LFP causality peak on the anti causal half-axis become wider, and the “shoulder” of that peak produced relatively high causality values at the negative half of the time lag axis as well. If only the instantaneous CCM were calculated, the IOSh → LFP causality coefficients would have similar values to the LFP → IOSh drive, thus one would erroneously conclude, that a significant feedback exists in the IOSh → LFP direction as well. Observation of the full, time dependent, cross-map function reveals, that the relatively high causality at zero time lag in the IOSh → LFP direction is only a side effect of a peak in the anti-causal regime. We concluded, that the delay dependent calculation of the CCM is important not only to determine the delays of the causal effects, but to find the correct causal directions and strengths as well.

The causality structure was very similar in all the three induction methods of the epileptic activity: electrical stimulation, *Mg*^2+^-free solution or 4AP induction, although the causal connection strength was stronger in the stimulated and weaker in the presence of 4AP than in *Mg*^2+^-free solution. The similarity of the causal structures may imply similar underlying mechanisms in all three cases, meaning, that the generation of the IOSh component is independent of the method of induction.

It was demonstrated, that although the cross-correlation functions showed peaks, those peaks did not reflect the actual causal dependency in these cases. Instead, the temporal derivative of the IOSh was correlated with the LFP power at the time delay of the actual causal peak.

During the interdischarge intervals, the IOSh signal decays towards the baseline exponentially, without significant causal influence from the LFP, which implies a linear autonomous dynamics.

To sum up our observations on the dynamics of IOSh, a simple model has been set up to describe the dependency of the IOSh on the LFP power. The model allowed the reconstruction of the IOSh based on the LFP signal. Model fitting showed, that during the slow development of the epileptiform activity, the effective causal connection strength slowly decreased. This decrease could be well approximated by an exponential decay. Quantitatively good reconstruction of IOSh, based on the LFP signal by our model during the 1 h long recordings, supports the results of the causality analysis as well.

The model provided us the possibility to cross-validate the causality analysis, with known causal dependency and effect delays. The causality analysis on the simulated data series resulted in very similar cross-map functions on the model as it was found on the measurements: the peaks on the negative half of the time delay axis precisely marked the direction and the delay of the simulated causal effects, while “shadow peaks” were generated on the positive, anti-causal half of the delay axis. The only slight difference was that in case of simulated data series, the shadow peaks position was always symmetric to the main peak on the delay axis, while in case of the measurements, the “shadow peak” showed larger lags.

Our results on LFP → IOSh unidirectional causality do not exclude the possible presence of causal connections on a slower time scale either in the reverse direction or in the same direction but between the observable epileptic bursts, where we now observe only the autonomous dynamics of the IOSh. Inference of possible interactions on slower time scale now excluded by filtering out the slower component (IOSl) which was necessary to make the IOSh available for the causal analysis.The causal relation between the LFP and the slower, IOSl component could not be determined based on these measurements, because the IOSl does not satisfy the necessary conditions. The application of the CCM method requires, that the system went through multiple times on its attractor. This condition is satisfied for the IOSh component, but not for the IOSl, which exhibits only one sweep through the state space, during these experiments.

The application of CCM for LFP and IOSh demonstrates, that the significantly different inherent speed and sampling rate of the signals, and the necessary downsampling does not preclude the determination of the causal relationships, thus raises the possibility of application of the new method to other signal modalities with different speed, such as fMRI and EEG as well.

## Methods

### Experimental procedures

Experiments were performed on adult, male Wistar rats weighing 100–200 g (Toxicoop, Budapest, Hungary). Experimental design conformed to the rules of European Communities Council Directive of 24 November 1986 (86/609/EEC), and the study was approved by the Animal Care and Use Committee of Eötvös Loránd University and Budapest Animal Health Care Authority (p.n.: 22.1/829/003/2007). Efforts were made to minimize the number of animals used. Rats were kept under a 12:12 h LD cycle (lights on at 8:00 a.m.) in a temperature-controlled room at 22 ± 2 °C. Standard food-pellets and tap water were available ad libitum.

### Slice preparation

*In vitro* experiments were performed on rat cortical slices (13 slices from 13 rats) using standard procedures. Animals were decapitated in deep chloral-hydrate (Hungaropharma, Budapest, Hungary) anesthesia and the brain was quickly removed from the skull. A block was dissected from the somatosensory cortex and 400 *μm* thick coronal slices were prepared with a vibratome (EMS-4000, Electron Microscopy Sciences, Fort Washington, PA, USA) in ice-cold artificial cerebrospinal fluid (ACSF). Slices were incubated at room temperature for an hour in oxygenated ACSF (pH 7.01–7.12), the composition of which was (in mM): 126 *NaCl*; 1.8 *KCl*; 1.25 *KH*_2_*PO*_4_; 1.3 *MgSO*_4_; 26 *NaHCO*_3_; 2.4 *CaCl*_2_; and 10 glucose.

### Induction and recording of seizure activity - interface conditions

A single slice was placed into an interface-type recording chamber, which was perfused continuously (3 ml/min) with ACSF. In the recording chamber, slices were maintained at 32 ± 0.5 °C in humidified, carbogenated (95%*O*_2_, 5%*CO*_2_) gas atmosphere. A total number of 12 recording sites in 12 slices were included in the final analysis. To test the change in the LFP, glass extracellular electrode (5–10 *M*Ω) filled with 1 M NaCl was positioned into layer 3 of the somatosensory cortex. For stimulation a bipolar tungsten electrode was placed at the border of the white and gray matter below the recording electrode (Fig. 1). Electrophysiological signals were amplified by an Axoclamp 2B amplifier (Axon Instruments Inc., Union City, CA), filtered and further amplified (0.16–1000 Hz, 1000x) by a Supertech Signal Conditioner (Supertech Ltd., Pécs, Hungary) and digitized by an NI-6023E A/D card (National Instruments, Austin, Texas) for off-line analysis. Intrinsic optical signals (IOS) were recorded also from the beginning of treatment with convulsant solution, simultaneously with local field potential (LFP). The slice was illuminated with unfiltered white light, using a voltage-stabilized cold light source (Fiber-Lite MI-150, Dolan-Jenner, Boxborough, UK). Alteration of reflected light was recorded by a monochrome 12-bit CCD camera (FOculus FO-432B, NET Gmbh, Lerchenberg, Germany) attached to an upright 3-way Olympus SZX-9 (Olympus, Tokyo, Japan) stereomicroscope. Digital images were taken at a resolution of 1024 × 768 pixels at 2 fps sampling rate, and stored in uncompressed format on an x86 based personal computer for off-line analysis Epileptiform discharges were provoked either by high frequency electric stimulation or by convulsant application into the perfusion solution. Evoked seizures developed as afterdischarges, which immediately followed the high frequency electric stimulation (50 Hz, 5 s). Spontaneous epileptiform activity developed after exchanging the perfusion solution in the recording chamber from ACSF to *Mg*^2+^-free- or 4-aminopiridine (4-AP, 50 *μM*) containing solution. These types of activity usually appeared in 10–25 min in following the perfusion of the convulsant.

Data acquisition of IOS and LFP was made simultaneously by a custom Matlab (The MathWorks Inc., Natick, MA, USA) based software, which made snapshots of continuous video signal from the CCD camera at a pre-defined sampling rate, based on the timing process of NI-6023E A/D card. For the analysis of the optical changes a region of interests (ROI) was manually defined in the slice beside the site of electrophysiological recording, optical alterations in a small box was determined (Fig. 1).

### Recording of seizure activity - submerged conditions

Under submerged conditions the recording of LFP was carried on a MEA USB-1060 INV (Multichannel Systems, Reutlingen, Germany) system. Slices were transferred to a 60 channel 200/30 3D multielectrode array (MEA) chip (Qwane Biosciences, Lausanne, Switzerland), where 30 µm diameter electrodes are positioned in a 8 × 8 grid of 200 µm distances. The slice is positioned directly on the bottom of MEA chip using a net, electrodes with the height of 50–70 Âµm penetrate into the lower surface of slice. Continuous ACSF perfusion was carried out at a 5–6 ml/min rate, temperature is maintained at 33 ± 1 °C using TC02 Temperature Controller (Multichannel Systems, Reutlingen, Germany) with PH01 perfusion heater (Multichannel Systems, Reutlingen, Germany) and the built-in heating pad of MEA 1060 Amplifier (Multichannel Systems, Reutlingen, Germany). The slice was trans illuminated with unfiltered white light, using a Rebel Star LED (Ledium Ltd, Szeged, Hungary) light source, placed under the MEA chip. Alteration of transmitted light was recorded by a 10-bit CCD camera (Qimaging Micropublisher 3.3, Qimaging, Surrey, Canada) attached to an upright 3-way Nikon SMZ 800 (Nikon Instruments Europe BV, Amsterdam, Netherlands) stereomicroscope. Digital images were taken at a resolution of 1024 × 768 pixels at 1 fps sampling rate, and stored in uncompressed format on an x86 based personal computer for off-line analysis Data acquisition of IOS and LFP was also made simultaneously in the case of submerged slice experiments. The STG4002 stimulator (Multichannel Systems, Reutlingen, Germany) provided continuous triggering for a custom Matlab (The MathWorks Inc., Natick, MA, USA) based framegrabber software during data acquisition made by MEA 1060 amplifier. Epileptic activity was provoked by *Mg*^2+^-free perfusion solution application.

### The intrinsic optical signals

Image series of interface and submerged slice experiments were analyzed with the same method, using a custom Mathworks Matlab based software. Since the first 2–3 min of video data acquisition was made in normal ACSF, the first 10 frames of each image series were averaged and served as control *F*_*cont*_, and then it was subtracted from each subsequent images according to the following formula:

5 $$IOS=\frac{{\rm{\Delta }}{F}_{t}}{{F}_{cont}}\cdot 100=\frac{{F}_{t}-{F}_{cont}}{{F}_{cont}}\cdot 100$$

where *F*_*cont*_ is the luminance of control image (the average of first 10 frames) at a given pixel, *F*_*t*_ is the luminance of each subsequent experimental image, Δ*F* is the change of reflectance or transmittance, depending on experimental conditions. *F*_*cont*_ in the denominator serves to normalize the data across regions differing in luminance. The images were converted to 256-scale pseudocolour images for further off-line analysis. For causality analysis a region of interests (ROI) was defined in the somatosensory cortical region of the slice around the site of electrophysiological recording. The mean IOS value was calculated for the ROI. This data was used to characterize the IOS changes of the slice during 1 h seizure induction and correlate with single-channel field potential recordings.

### Convergent Cross Mapping

The CCM algorithm is based on the Takens’ theorem^25^, which states, that time delay embedding of an observed time series makes possible the reconstruction of the attractor of a dynamical system, up to a topological equivalence. Here we used a time-symmetric embedding, which makes possible more precise identification of the effect’s delay:

6 $${\bf{X}}(t)=[X(t-D\tau ),\,\mathrm{...,}\,X(t-2\tau ),X(t-\tau ),X(t),X(t+\tau ),X(t+2\tau )\,\mathrm{...,}\,X(t+D\tau )]$$

where *X*(*t*) denotes the original time series, ***X***(*t*) is the time series of the embedded *E* = 2D + 1 dimensional vectors and *τ* is the delay used for the embedding.

The CCM algorithm embeds and reconstructs the attractors of the two investigated time series and tests whether the “fingerprint” of the cause can be observed in the consequence. To infer the causality from *X*(*t*) to *Y*(*t*) time series, the CCM algorithm runs through all the points **Y**(*t*) of the consequence. For all **Y**(*t*) points of the manifold, the *E* + 1 nearest neighbor points **Y**(*t*_*i*_) are chosen. A weighting factor *W*_*i*_(*t*) is calculated for each neighbor **Y**(*t*_*i*_) through: 1, normalizing the distances by the distance of the closest neighbor **Y**(*t*_1_); 2, using an exponential kernel; 3, renormalizing to sum 1:

7 $${W}_{i}(t)=\frac{{e}^{-\frac{|{\bf{Y}}({t}_{i})-{\bf{Y}}(t)|}{|{\bf{Y}}({t}_{1})-{\bf{Y}}(t)|}}}{\sum _{i}^{E+1}{e}^{-\frac{|{\bf{Y}}({t}_{i})-{\bf{Y}}(t)|}{|{\bf{Y}}({t}_{1})-{\bf{Y}}(t)|}}}$$

where, $|{\bf{Y}}({t}_{i})-{\bf{Y}}(t)|$ denotes the Euclidean distance between the *i*^*th*^ closest neighbor **Y**(*t*_*i*_) and the original point **Y**(*t*) in the embedding space. Then, an estimation $\hat{X}(t)$ is calculated for *X*(*t*) as a weighted average of the mapped *X*(*t*_*i*_) points, by using the *W*_*i*_(*t*) weight factors:

8 $$\hat{X}(t)=\sum _{i}{W}_{i}(t)\,\ast \,X({t}_{i})$$

Finally, the estimated $\hat{X}(t)$ and the original *X*(*t*) time series are compared by calculating the linear correlation coefficient between them:

9 $$CC{M}_{X\to Y}=\frac{{\sum }_{t}(X(t)-M(X(t)))(\hat{X}(t)-M(\hat{X}(t)))}{\sqrt{\sum _{t}{(X(t)-M(X(t)))}^{2}}\sqrt{\sum _{t}{(\hat{X}(t)-M(\hat{X}(t)))}^{2}}}$$

where *M*(*X*(*t*)) denotes the mean of *X*(*t*) over the time samples. High values of the *CCM*_*X*→*Y*_ show, that the consequence *Y*(*t*) contains information about the cause *X*(*t*), thus refers to the existence of a directed causal connection from the cause to the consequence. Causality *CCM*_*Y*→*X*_ in the reverse direction calculated similarly, only the role of the *X* and *Y* is reversed.

Ye *et al*.^13^ extended the original CCM method by introducing time delay between the cause and consequence and examining the causal relationships at many time lags.

Theoretically, if the dimension of the dynamics is *D*, than at most an *E* = 2*D* + 1 dimensional embedding space is needed to reach the full topological equivalence between the embedded and the original attractors^25^. However, *E* = *D* + 1 embedding dimension is enough, to turn the false self-cross sections of the manifold into zero-volume^26^. In practice, we found that higher embedding dimension and higher *τ* embedding delay resulted in increasing causality-peak for a while, but widening plateau on the top of the peak after a certain point. Thus, we tried to find parameter set, which is optimal for the inference of the effect’s temporal delay, which results in the highest peak without plateau on it. As a result, the embedding dimension was chosen to 3 and the time delay for the embedding to 1 time step (0.5 s) for the analysis.

## Data Availability

The datasets generated during and analyzed during the current study are available from the corresponding author on request.
